# Tacrolimus alleviates LPS‐induced AKI by inhibiting TLR4/MyD88/NF‐κB signalling in mice

**DOI:** 10.1111/jcmm.17108

**Published:** 2021-12-09

**Authors:** Xueqing Hu, Wenqian Zhou, Shun Wu, Rui Wang, Zhiyong Luan, Xin Geng, Na Xu, Zhaoyong Zhang, Zhenmin Ruan, Zenghui Wang, Furong Li, Chen Yu, Hongqi Ren

**Affiliations:** ^1^ Department of Nephrology the Affiliated Huaihai Hospital of Xuzhou Medical University Xuzhou China; ^2^ Department of Nephrology Tongji Hospital School of Medicine, Tongji University Shanghai China; ^3^ Department of Nephrology Xinqiao Hospital Army Medical University (Third Military Medical University) Chongqing China

**Keywords:** acute kidney injury, lipopolysaccharide, podocyte, Toll‐like receptor 4

## Abstract

Lipopolysaccharide (LPS)‐induced sepsis‐associated acute kidney injury (SA‐AKI) is a model of clinical serious care syndrome, with high morbidity and mortality. Tacrolimus (TAC), a novel immunosuppressant that inhibits inflammatory response, plays a pivotal role in kidney diseases. In this study, LPS treated mice and cultured podocytes were used as the models of SA‐AKI *in vivo* and *in vitro*, respectively. Medium‐ and high‐dose TAC administration significantly attenuated renal function and renal pathological manifestations at 12, 24 and 48 h after LPS treatment in mice. Moreover, the Toll‐like receptor 4 (TLR4)/myeloid differential protein‐88 (MyD88)/nuclear factor‐kappa (NF‐κB) signalling pathway was also dramatically inhibited by medium‐ and high‐dose TAC administration at 12, 24 and 48 h of LPS treatment mice. In addition, TAC reversed LPS‐induced podocyte cytoskeletal injury and podocyte migratory capability. Our findings indicate that TAC has protective effects against LPS‐induced AKI by inhibiting TLR4/MyD88/NF‐κB signalling pathway and podocyte dysfunction, providing another potential therapeutic effects for the LPS‐induced SA‐AKI.

## 1 INTRODUCTION

Sepsis is a systemic inflammatory response caused by infection,^1^, ^2^ which most often leads to multiorgan dysfunction, and kidney dysfunction is defined as sepsis‐associated acute kidney injury (SA‐AKI) under this condition. SA‐AKI accounts for about 50% cases of acute kidney injury (AKI) in ICU, and the hospital mortality of SA‐AKI patients is extremely high.^3^, ^4^ Hence, it is essential to explore effective therapeutic agents to reduce the mortality of SA‐AKI in patients.

Lipopolysaccharide (LPS), produced by Gram‐negative bacteria, is one of the most common molecules to induce SA‐AKI.^5^, ^6^ Toll‐like receptor 4 (TLR4) is the most vital receptors for LPS, and involves in the pathogenesis of acute renal injury and chronic kidney diseases, including diabetic nephropathy, ischaemia reperfusion AKI and SA‐AKI.^6^, ^7^, ^8^, ^9^ Upon ligand binding, TLR4 induces the activation of nuclear factor‐kappa (NF‐κB) via recruiting myeloid differential protein‐88 (MyD88), leading to inflammatory response, oxidative stress and cell apoptosis, and eventually result in renal dysfunction in mice model of SA‐AKI.^9^, ^10^ In addition, inhibition of TLR4 signalling protects against SA‐AKI development in mice.^11^, ^12^ Thus, agents that are capable of suppressing TLR4 signalling might be effective on treating SA‐AKI.

Tacrolimus (TAC) is an effective immunosuppressant. It acts to bind to FKBP12 and in turn prevents calcium‐induced calcineurin activation, which inhibits the induction of inflammatory cytokines.^13^, ^14^ TAC is exploited in patients with transplant rejection and autoimmune diseases, including systemic lupus erythematosus and autoimmune hepatitis.^15^, ^16^, ^17^ Meanwhile, studies have demonstrated that TAC can alleviate proteinuria, podocyte injury and further renal dysfunction.^18^, ^19^, ^20^ However, whether TAC is effective for SA‐AKI has not been explored. In this study, we demonstrate that TAC is capable of alleviating LPS‐induced SA‐AKI through inhibiting the TLR4/MyD88/NF‐κB signalling pathway and podocyte dysfunction.

## 2 MATERIALS AND METHODS

### 2.1 Reagents

The reagents used in current study include: LPS (Sigma, Chemical Co.), Tacrolimus (Astellas), foetal bovine serum (ExCell Biology), RPMI 1640 culture medium, penicillin and streptomycin solution, 0.5% Trypsin‐EDTA (Gibco), Phalloidin (Sigma, Chemical Co.), RIPA Lysis buffer, beta‐actin antibody (Beyotime Biotechnology), protein buffer (Beyotime Biotechnology), p65, phospho‐p65 (pp65) antibody (Cell Signaling Technology), MyD88 antibody (Boosen) and TLR4 antibody (Abcam).

### 2.2 Animal model of LPS nephrosis

Sixty male C57BL/6 mice (Laboratory Animals Center of Xuzhou Medical College, Xuzhou, China) were housed in cages at 24 to 26°C with alternating 12 h light/dark cycles, and had free access to regular food and water. Mice were divided into five groups: normal control (NC) group (*n* = 12), LPS model group (*n* = 12) and low‐ (*n* = 12), medium‐ (*n* = 12) and high‐dose (*n* = 12) TAC treatment groups. Except for NC group, mice were injected intraperitoneally with 200 μg LPS, which dissolved into 200 μL sterile saline. NC group received the same volume of sterile saline. After LPS injection, the mice were injected with TAC (low dose of 1 mg/kg, medium dose of 2 mg/kg and high dose of 4 mg/kg per 24 h); treatment continued throughout the experiment. The study was approved by the Committee on Ethical Use of Animals of Xuzhou Medical College.

### 2.3 Mouse sacrifice and kidney tissue processing

At 12, 24 and 48 h after LPS treatment, mice were sacrificed under anaesthesia with 1% pentobarbital. We collected the kidney tissues, which were processed for standard histological examination, including fixation in 10% formalin, dehydration in graded alcohol solution, embedding in paraffin, etc. The renal cortex tissue was also used for immunoblotting assay after being lysed with RIPA buffer as described below.

### 2.4 Blood samples

At 12, 24 and 48 h after LPS treatment, blood samples were collected for biochemical analysis. All samples were kept at –80°C, blood urea nitrogen (BUN) and creatinine levels were measured using Nanjing Jiancheng Urea Assay kit and Creatinine (Cr) Assay kit (sarcosine oxidase).

### 2.5 Histological examination

Two‐micrometre‐thick sections were cut and stained with haematoxylin and eosin and periodic acid‐Schiff reagent, respectively, following the methods described.^7^ All slides were imaged by an Olympus microscope and the images were evaluated by the same pathologist.

### 2.6 Immunohistochemistry of TLR4, MyD88 and pp65

Deparaffinized 4 μm sections were microwaved at 95°C for 20 min in 0.01 mol/L citric acid buffer (pH 6.0) and then incubated with 3% H_2_O_2_ for 15 min to consume endogenous peroxidase. After washing with PBS, the sections were incubated with anti‐TLR4 antibody (1:200; Abcam), anti‐MyD88 antibody (1:200, Boosen) and anti‐p65 antibody (1: 200; Cell Signaling Technology) for 2 h at 37°C. After washing with PBS, sections were incubated with HRP‐labelled goat anti‐rabbit secondary antibody (1: 1,000; Santa Cruz) for 30 min at 37°C. Finally, diaminobenzidine substrate (ZSGB‐BIO) was used to visualize positive areas.

### 2.7 Western blot

The renal tissues were dissolved in RIPA lysis buffer (Beyotime Biotechnology) supplemented with protease inhibitors and its concentration was measured with a BCA kit (Beyotime Biotechnology). The protein samples were added to sodium dodecyl sulphate–polyacrylamide gel and separated by electrophoresis, and the then separated protein is transferred to polyvinylidene difluoride membranes. Polyvinylidene difluoride membranes were immersed in 5% non‐fat milk for 2 hours to block non‐specific antigen and then incubated overnight at 4℃ with the primary antibody, including anti‐TLR4 antibody (1:500; Abcam), anti‐ MyD88 antibody (1:300; BIOSS, China), anti‐p65 antibody (1:1000; Cell Signaling Technology), anti‐pp65 antibody (1:1000; Cell Signaling Technology) and anti‐beta‐actin (1:1000; Beyotime Biotechnology). After washing with TBST, the membranes were then incubated with the second antibody for 1 h at room temperature. Finally, the target proteins were detected by highly sensitive ECL reagent (Bio‐Rad Laboratories), and exposed in the BIO‐RAD Chemical Imaging System.

### 2.8 RT‐qPCR

The renal tissues were dissolved in RNAiso Plus Reagent (TaKaRa), and the RNA was extracted. Then, cDNA was synthesized according to M‐MLV Reverse Transcriptase kit (Invitrogen, USA). Finally, qRT‐PCR was performed using SYBR Green I kit (TaKaRa) by Applied Biosystems 7500. The primers sequences were as follows: beta‐actin forward, 5′‐GGGAAATCGTGCGTGACATTAAG‐3′, reverse, 5′‐TGTGTTGGCGTACAGGTCTTTG‐3′; TLR4 forward 5′ ‐CAAGAACATAGATCTGAGCTTCAACCC‐3′, reverse, 5′‐GCTGTCCAATAGGGAAGCTTTCTAGAG‐3′; MyD88 forward, 5′‐AAGAAAGTGAGTCTCCCCTC‐3′, reverse, 5′‐TCCCATGAAACCTCTAACAC‐3′; p65 forward, 5′‐GCTCGGCTGAATGAATCTACC‐3′ and reverse, 5′‐GTCTCCACGTATTTCCGCAACT‐3′. The thermal cycling for amplification included denaturation at 94 °C, followed by 40 cycles of 30 s at 94°C, 34 s at 60°C and 30 s at 72°C. After the last cycle, incubation at 72°C for 10 min was followed. These experiments were repeated three times independently. All data were normalized by beta‐action levels.

### 2.9 Podocyte culture and treatment

The immortalized mouse podocyte cell line was obtained from the National Clinical Research Center for Kidney Diseases (Nanjing, Jiangsu) and stored in the Department of Laboratory of Immunity and Metabolism (Xuzhou Medical College). The podocytes at passages 19 to 25 were used in all experiments. The cells were initially cultured in 1640 media (Hyclone, USA) supplemented with 10% foetal bovine serum (ExCell Biology), 100 U/mL penicillin and 100 μg/mL streptomycin (Gibco, USA) on Primaria plates at 33°C in 5% CO_2_. To induce the development of the differentiated and quiescent phenotype, the cells were grown at 37°C for 14 days, and these differentiated cells were used for all experiments. Cells were divided into five groups: NC group, LPS group, low‐dose TAC treatment, medium‐dose TAC treatment and high‐dose TAC treatment group. Except for NC group, the cells were exposed to LPS (25 μg/ml) for 6 h and then incubated in medium containing 0.1% DMSO. In the low‐, medium‐ and high‐dose TAC group, cells were treated with TAC (low dose of 0.5 μg/ml, medium dose of 1μg/mL and high dose of 2 μg/ml); treatment continued throughout the experiment. NC group cells were grown in the control medium. Cells were collected after TAC treatment for 6, 12 and 24 h. All experiments were repeated at least three times for each indicated condition.

### 2.10 Phalloidin staining for F‐actin

Mouse podocytes growing on type I collagen‐coated (Sigma‐Aldrich) glass slides were pretreated with different conditions and then fixed in 4% paraformaldehyde for 10 min followed by being permeabilized in 0.2% Triton ×‐100 for 10 min. Phalloidin (Sigma‐Aldrich) was used to stain F‐actin in the podocytes, and the resulting images were examined by an inverted fluorescent microscope (Olympus).

### 2.11 Wound healing assays

Mouse podocyte pretreated with different conditions was allowed to grow to confluence on type I collagen‐coated (Sigma‐Aldrich) culture dishes. Cell monolayers were washed and scratch wounds were applied using a 200 μl pipet tip. Podocytes were imaged using microscope at time 0 immediately after wound creation. Cells were then returned to non‐permissive growth conditions for 24 h before final imaging of wound healing.

### 2.12 Transwell migration assay

Transwell cell culture inserts (Corning) were coated with type I collagen (Sigma‐Aldrich), rinsed once with PBS and placed in RPMI medium in the lower compartment. About 1 × 10^4^/ml podocytes from each group were seeded in the inserts and then allowed to migrate for 24 h at 37°C. Non‐migratory cells were removed from the upper surface of the membrane and the migrated cells were fixed with 4% paraformaldehyde and stained with haematoxylin. The number of migrating cells was counted using phase contrast microscopy (Leica).

### 2.13 Statistical analysis

Statistical analyses were performed with SPSS 16.0 software (version 16.0, SPSS Inc.). The results were expressed as the mean ± SD. Statistical analysis of the differences between two groups of treatment was performed using variance analysis combined with the rank sum test. *p* < 0.05 was considered statistically significant.

## 3 RESULTS

### 3.1 Renal function of the mice treated with LPS and TAC

**FIGURE 1:**
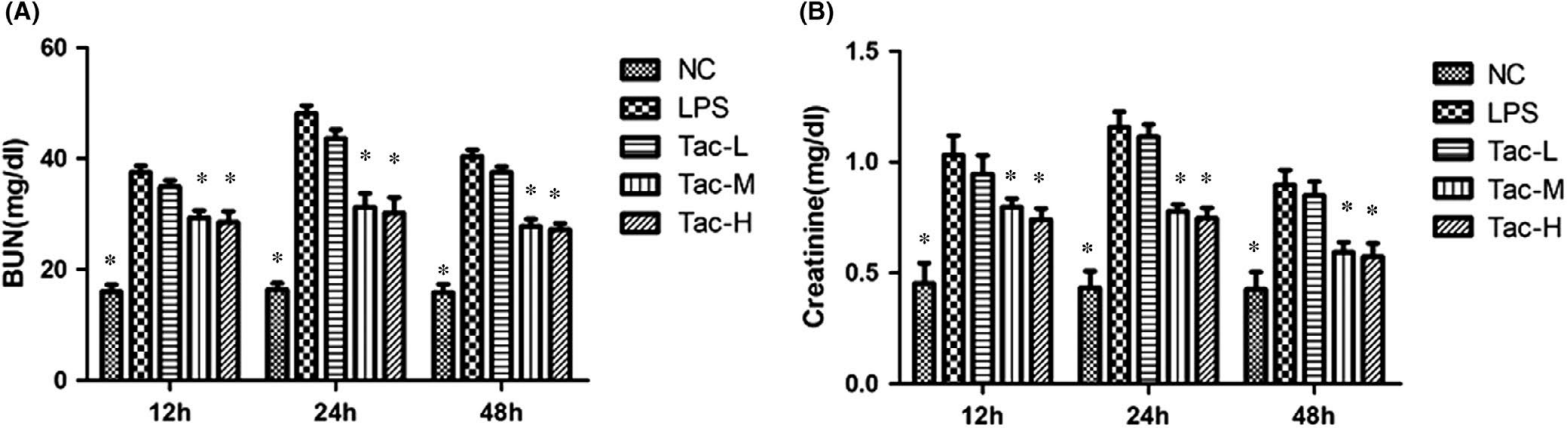
Effect of TAC on LPS‐induced renal dysfunction in mice. (A) BUN and (B) Creatinine. NC, normal control group; LPS, LPS model group; Tac‐L, low‐dose TAC treatment group; Tac‐M, medium‐dose TAC treatment group; Tac‐H, high‐dose TAC treatment group. *p < 0.05 vs. LPS

To analyse kidney function, BUN and serum creatinine were determined. As shown in Figure 1, LPS induced significant increases in both BUN and serum creatinine levels, and the increases were significantly attenuated by medium‐ and high‐dose TAC treatment.

### 3.2 Pathological Findings in mice treated with LPS and TAC

**FIGURE 2:**
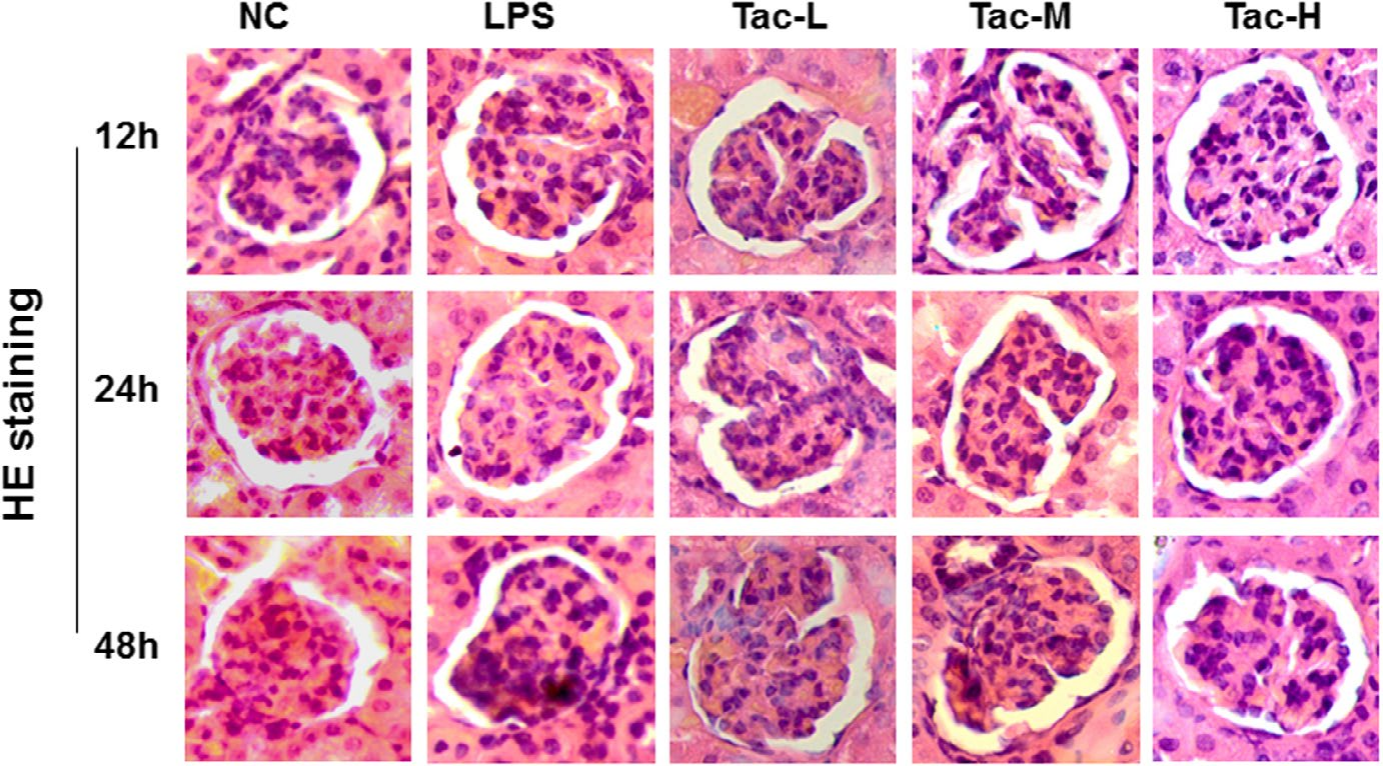
Haematoxylin and eosin staining of kidney section from the mice treated with LPS and TAC. Original magnification ×400. NC, normal control group; LPS, LPS model group; Tac‐L, low‐dose TAC treatment group; Tac‐M, medium‐dose TAC treatment group; Tac‐H, high‐dose TAC treatment group

The light microscopy with haematoxylin and eosin staining revealed that LPS treatment caused inflammatory cells infiltration, glomerular capillary congestion, mesangial cells proliferation, mesangial matrix expansion, focal segmental glomerulosclerosis, capsule adhesion, glomerular basement membrane abnormality and the pathological changes were reversed by medium‐ and high‐dose TAC treatment (Figure 2).

### 3.3 TLR4, MyD88 and p65 expression examination by IHC

**FIGURE 3:**
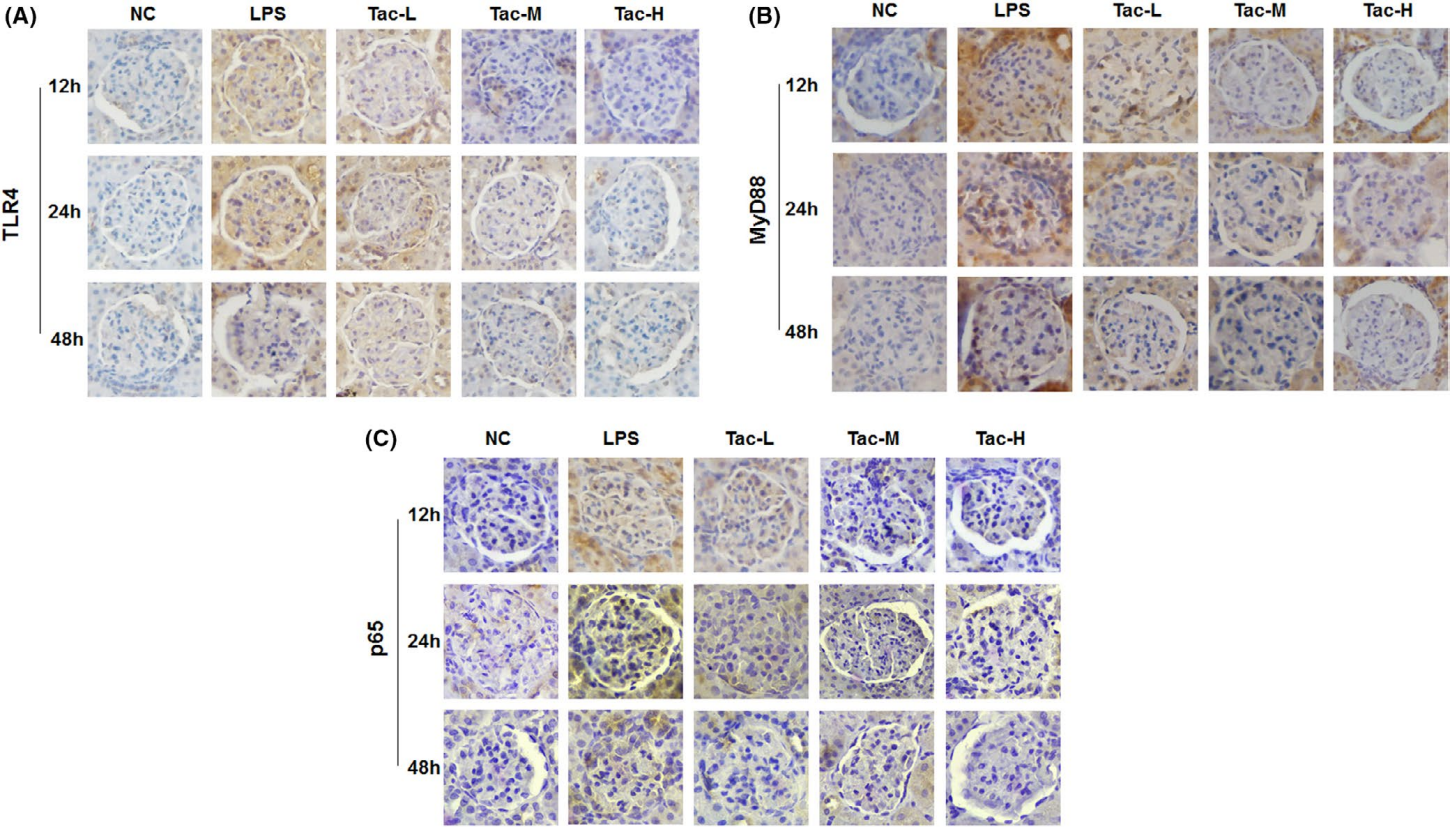
IHC staining of TLR4, MyD88 and p65 in the mice treated with LPS and TAC. (A) TLR4; (B) MyD88 and (C) p65. NC, normal control group; LPS, LPS model group; Tac‐L, low‐dose TAC treatment group; Tac‐M, medium‐dose TAC treatment group; Tac‐H, high‐dose TAC treatment group

As shown in Figure 3, IHC studies revealed that LPS treatment upregulated the expression of TLR4, MyD88 and p65 in glomeruli of the mice, and treatment with the medium‐dose and high‐dose TAC prevented the upregulation of TLR4, MyD88 and p65 expression.

### 3.4 TLR4, MyD88 and pp65/p65 expression examination by Western blotting

**FIGURE 4:**
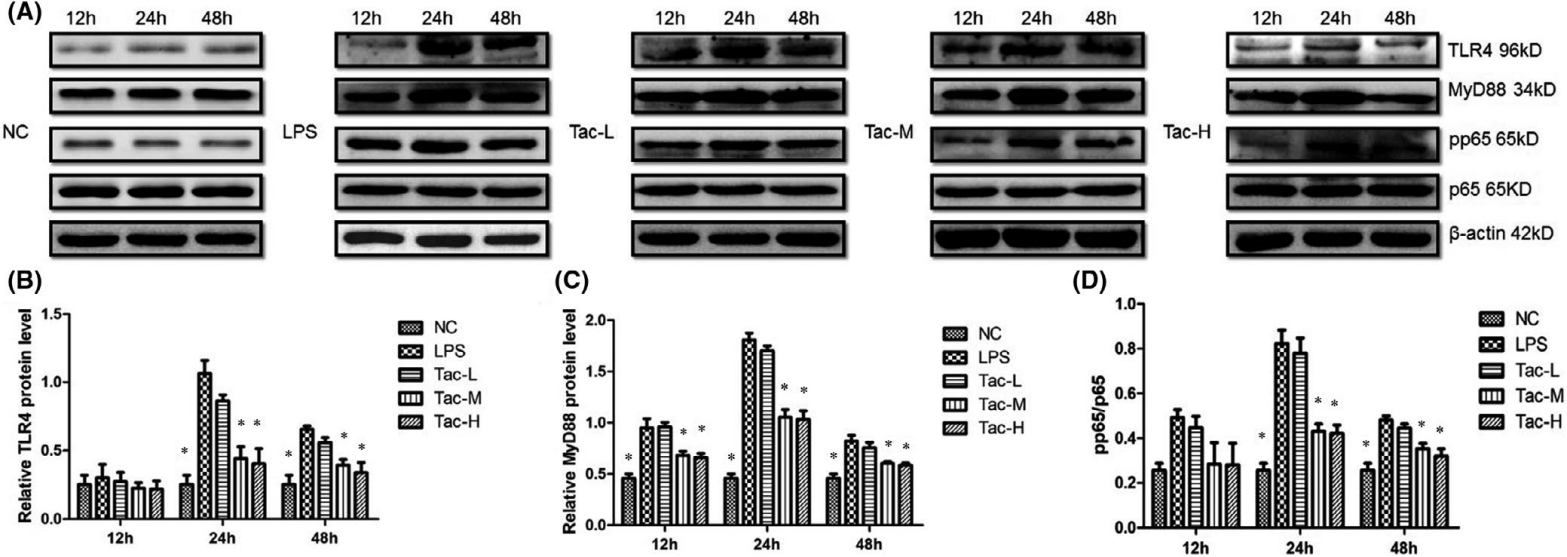
The protein expression levels of TLR4, MyD88 and pp65/p65 in mice treated with LPS and TAC. (A) Representative Western blotting images of TLR4, MyD88, p65 and pp65. (B) Quantification of the TLR4; (C) quantification of the MyD88 and (D) quantification of the pp65/p65. NC, normal control group; LPS, LPS model group; Tac‐L, low‐dose TAC treatment group; Tac‐M, medium‐dose TAC treatment group; Tac‐H, high‐dose TAC treatment group; *p < 0.05 vs. LPS

To perform quantitative analysis of TLR4, MyD88 and pp65/p65 expression changes in kidney tissues of different groups of mice, Western blotting was performed. The results showed LPS significantly upregulated protein levels of TLR4, MyD88 and pp65/ p65 in renal tissues, and TAC remarkably attenuated the upregulation of TLR4, MyD88 and pp65/p65, as shown by the fact that medium‐ and high‐dose TAC resulted in a significantly lower levels of these proteins compared with that of the LPS group (Figure 4).

### 3.5 qRT‐PCR analysis of TLR4, p65 and MyD88

**FIGURE 5:**
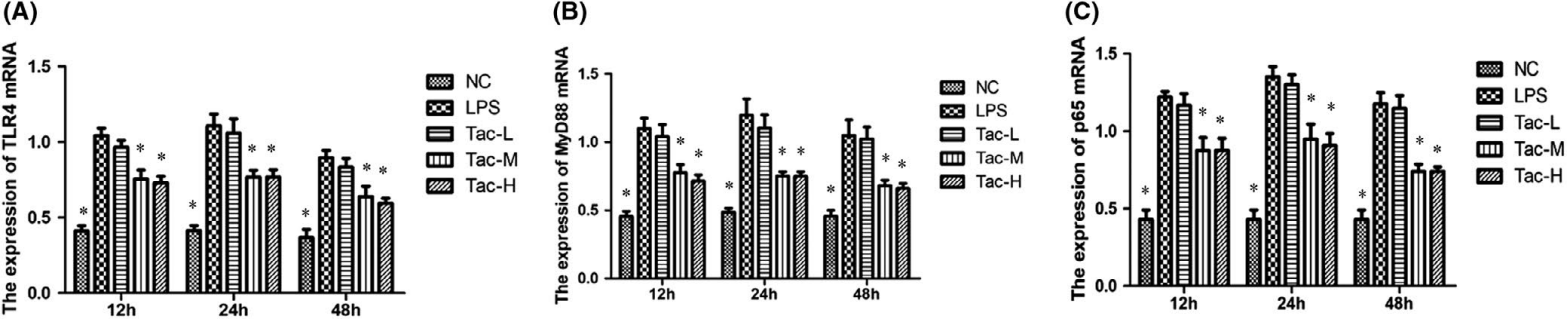
The mRNA levels of TLR4, MyD88 and p65 in the mice treated with LPS and TAC. (A) TLR4; (B) MyD88 and (C) p65. NC, normal control group; LPS, LPS model group; Tac‐L, low‐dose TAC treatment group; Tac‐M, medium‐dose TAC treatment group; Tac‐H, high‐dose TAC treatment group; *p < 0.05 vs. LPS

According to the qRT‐PCR results, LPS significantly upregulated the mRNA levels of TLR4, MyD88 and p65 in kidney tissues, and the mRNA levels upregulation was attenuated by the medium‐ and high‐dose TAC treatments, and the medium‐ and high‐dose TAC groups did not exhibit any difference in preventing upregulation of these proteins (Figure 5).

### 3.6 Cytoskeletal analysis of cultured podocytes treated with LPS and TAC

**FIGURE 6:**
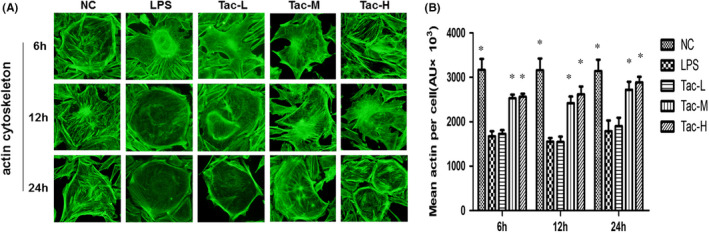
TAC reversed LPS‐induced podocyte actin cytoskeleton injury. (A) Phalloidin staining of cultured podocytes treated with LPS and TAC as indicated (original magnification ×1000); and (B) quantification of the F‐actin stress fibres expression. NC, normal control group; LPS, LPS model group; Tac‐L, low‐dose TAC treatment group; Tac‐M, medium‐dose TAC treatment group; Tac‐H, high‐dose TAC treatment group

As shown in Figure 6, podocytes under the normal culture condition displayed a regular filamentous arrangement of continuous actin fibres, and LPS caused a loss, disarrangement and disruption of the fine F‐actin stress fibres in the cells after 6h exposure with TAC treatment. Also, the number, morphology and distribution of F‐actin stress fibres were restored by medium‐ and high‐dose TAC treatments. In contrast, low‐dose TAC treatment had no such effects (Figure 6).

### 3.7 The wound healing and transwell experiments

**FIGURE 7:**
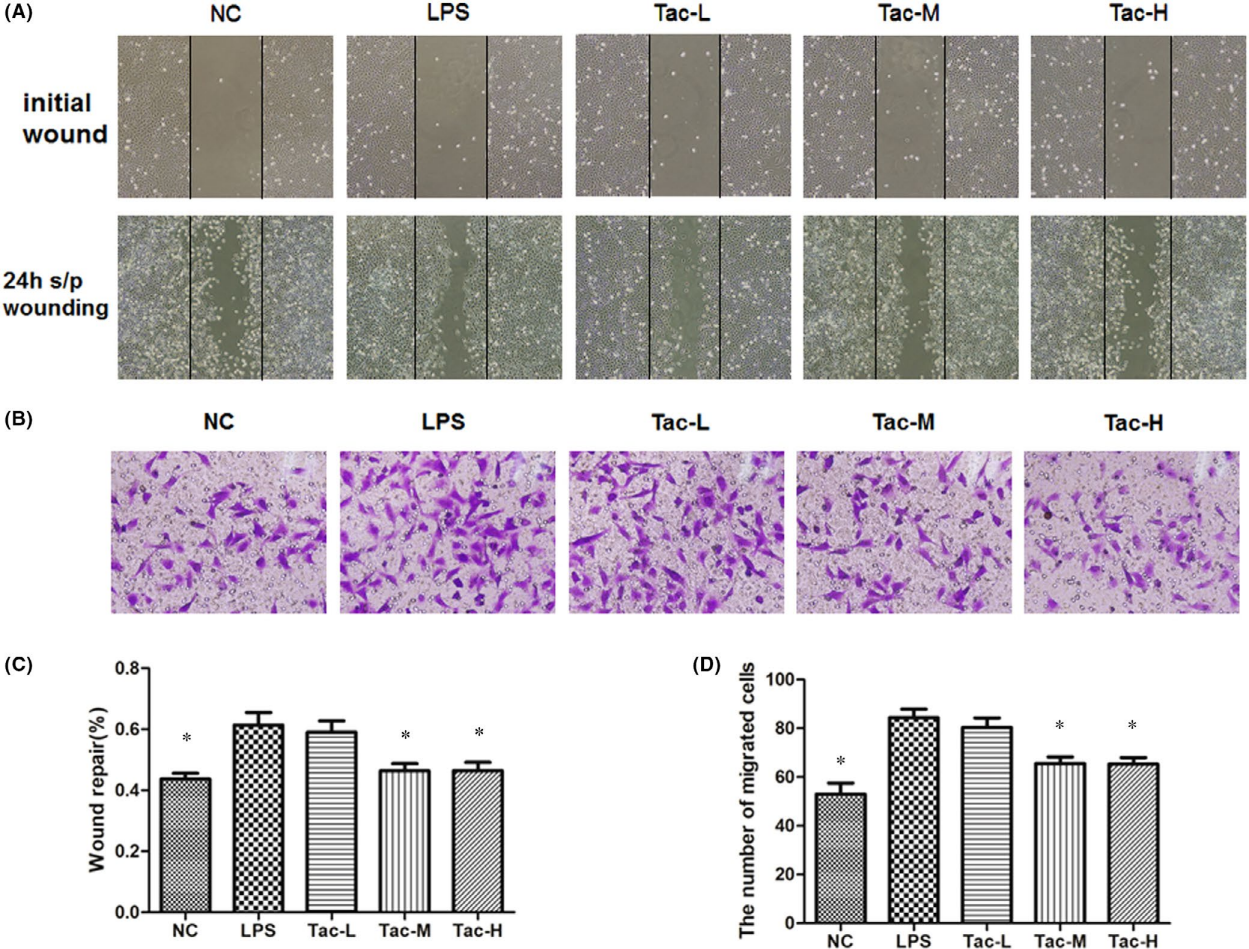
Podocyte motility measurement by wound healing assay and transwell migration assay. (A) Wound healing assay; (B) modified transwell migration assay; (C) quantification of the wound healing assay and (D) quantification of modified transwell migration assay. NC, normal control group; LPS, LPS model group; Tac‐L, low‐dose TAC treatment group; Tac‐M, medium‐dose TAC treatment group; Tac‐H, high‐dose TAC treatment group, *p < 0.05 vs. LPS

The wound healing and transwell experiments both showed that the migration of podocytes in the LPS group and low‐dose TAC group was faster than that of the normal group. However, podocyte migration in the medium‐ and high‐dose TAC groups was much slower than that of the LPS group (Figure 7), indicating that TAC inhibited LPS‐induced increase in podocyte migratory capability.

## 4 DISCUSSION

SA‐AKI has shown high hospital mortality in patients, and LPS is one of the most common causes of this disease.^3^, ^4^, ^5^ Therefore, there is an urgent need for appropriate therapeutic drugs to halt the progression of LPS‐induced SA‐AKI. As a novel immunosuppressant, TAC has been reported to have the effect to alleviate proteinuria, podocyte abnormality and renal dysfunction in acute and chronic kidney diseases, including renal allograft rejection, IgA nephropathy and diabetic nephropathy.^7^, ^8^, ^9^, ^20^ To our best knowledge, the effects and exact mechanisms of TAC in the pathogenesis of LPS‐induced SA‐AKI have not yet been investigated. In the present study, we used mice with intraperitoneal injection of LPS as a model of SA‐AKI, and found that LPS treatment induced renal dysfunction and renal pathological changes, which was consistent with previous studies.^11^, ^21^ In addition, medium‐ and high‐dose TAC alleviated the development of SA‐AKI in mice at 12h, 24h and 48h after LPS treatment, as evidenced by the lowered levels of BUN and serum creatinine, and improved renal pathological changes, suggesting that TAC (dose more than 2 mg/kg) could be a novel treatment for LPS‐induced SA‐AKI.

TLR4 is able to recognize LPS and triggers multiple signalling pathways for inflammatory response in the cells, eventually leading to cellular damage.^22^ In addition, an increasing body of evidence demonstrates that TLR4 involved in the development of LPS‐induced SA‐AKI and TLR4 inhibition (TLR4 inhibitor TAK‐242 or TLR4 gene knockout) reduces inflammatory response and cellular damage, improves glomerular filtration rate and urine output, and further decreases endotoxaemia‐associated mortality in the animal models of LPS‐induced SA‐AKI.^23^, ^24^ Consistent with the results from animal studies, TLR4 inhibitors also exhibit renoprotective effect in the patients with SA‐AKI.^25^ Thus, TLR4 may be a potential target for treating SA‐AKI. In the phase of TLR4 activation, MyD88 recruits to TLR4, and subsequently mediates the IKK complex phosphorylation, resulting in nuclear NF‐κB translocation and proinflammatory genes expression. In line with previous studies,^22^ we found that TLR4 and MyD88 expression was significantly upregulated and NF‐κB signalling pathway was activated in the kidney of the mice with LPS‐induced SA‐AKI. In addition, our results showed that medium‐ and high‐dose TAC prevented the upregulation of TLR4 and MyD88, and the activation of NF‐κB signalling pathway in the development of SA‐AKI in mice at 24h and 48h after LPS treatment, suggesting that TAC (dose more than 2 mg/kg) exerts renoprotective effect through TLR4/MyD88/NF‐κB pathway in LPS‐induced SA‐AKI mice.

Considering the critical role of podocytes in the development of LPS‐induced SA‐AKI,^26^, ^27^ we further investigated the effect of TAC in LPS‐induced podocyte injury, and found that medium‐ and high‐dose TAC reversed LPS‐induced podocyte cytoskeletal injury and podocyte migratory capability, indicating that the protective effect of TAC against LPS‐induced SA‐AKI may attribute to its capability to restore podocyte structure and function.

In conclusion, this is the first report demonstrating that TAC has protective effects on LPS‐induced AKI by inhibiting TLR4/MyD88/NF‐κB signalling pathway and preventing podocyte dysfunction. Therefore, TAC may be a potential therapeutic drug for treating LPS‐induced SA‐AKI in patients.

Some limitations of this study should be noted. Firstly, p65 expression was shown to be upregulated by LPS by IHC and qRT‐PCR, while it appears not changed by WB. We are confused with this phenomena and further studies should be performed. Secondly, we have only studied the effects of TAC on TLR4/MyD88/NF‐κB signalling pathway *in vivo* and podocyte function damage *in vitro* respectively. Further studies are required to determine the mechanisms and relationship between TLR4/MyD88/NF‐κB signalling pathway and podocyte dysfunction in the development of LPS‐induced SA‐AKI. Thirdly, TLR4 can activate at least two signalling pathways: the MyD88‐dependent pathway (TLR4/MyD88/NF‐κB pathway) and the TRIF‐dependent pathway. We have only explored TLR4/MyD88/NF‐κB signalling pathway, and left TRIF‐dependent pathway unexplored. It would be important to investigate the potential role of TRIF‐dependent pathway in the action of TAC in further study.

## CONFLICTS OF INTEREST

The authors declare no conflict of interest.

## AUTHOR CONTRIBUTIONS

**Xueqing Hu:** Data curation (equal); Investigation (equal); Methodology (equal); Software (equal); Writing – review & editing (equal). **Wenqian Zhou:** Conceptualization (equal); Formal analysis (equal); Funding acquisition (equal). **Shun Wu:** Data curation (equal); Investigation (equal); Methodology (equal); Software (equal); Writing – original draft (equal). **Rui Wang:** Data curation (equal); Software (equal). **Zhiyong Luan:** Data curation (equal); Software (equal). **Xin Geng:** Data curation (equal); Software (equal). **Na Xu:** Data curation (equal); Software (equal). **Zhaoyong Zhang:** Data curation (equal); Software (equal). **Zhenmin Ruan:** Data curation (equal); Software (equal). **Zenghui Wang:** Data curation (equal); Software (equal); Supervision (equal). **Furong Li:** Writing – review & editing (equal). **Chen Yu:** Investigation (equal). **Hongqi Ren:** Conceptualization (equal); Data curation (equal); Formal analysis (equal); Funding acquisition (equal); Investigation (equal); Methodology (equal); Project administration (equal); Software (equal); Supervision (equal); Visualization (equal); Writing – original draft (equal); Writing – review & editing (equal).

## Data Availability

Encourages Data Sharing.
